# Male urine signals social rank in the Mozambique tilapia (*Oreochromis mossambicus*)

**DOI:** 10.1186/1741-7007-5-54

**Published:** 2007-12-12

**Authors:** Eduardo N Barata, Peter C Hubbard, Olinda G Almeida, António Miranda, Adelino VM Canário

**Affiliations:** 1 CCMAR-CIMAR Laboratório Associado, Universidade do Algarve, Campus de Gambelas, 8005-139 Faro, Portugal; 2 Departamento de Biologia, Universidade de Évora, Apartado 94, 7002-554 Évora, Portugal

## Abstract

**Background:**

The urine of freshwater fish species investigated so far acts as a vehicle for reproductive pheromones affecting the behaviour and physiology of the opposite sex. However, the role of urinary pheromones in intra-sexual competition has received less attention. This is particularly relevant in lek-breeding species, such as the Mozambique tilapia (*Oreochromis mossambicus*), where males establish dominance hierarchies and there is the possibility for chemical communication in the modulation of aggression among males. To investigate whether males use urine during aggressive interactions, we measured urination frequency of dye-injected males during paired interactions between size-matched males. Furthermore, we assessed urinary volume stored in the bladder of males in a stable social hierarchy and the olfactory potency of their urine by recording of the electro-olfactogram.

**Results:**

Males released urine in pulses of short duration (about one second) and markedly increased urination frequency during aggressive behaviour, but did not release urine whilst submissive. In the stable hierarchy, subordinate males stored less urine than males of higher social rank; the olfactory potency of the urine was positively correlated with the rank of the male donor.

**Conclusion:**

Dominant males store urine and use it as a vehicle for odorants actively released during aggressive disputes. The olfactory potency of the urine is positively correlated with the social status of the male. We suggest that males actively advertise their dominant status through urinary odorants which may act as a 'dominance' pheromone to modulate aggression in rivals, thereby contributing to social stability within the lek.

## Background

Pheromones are involved in the reproductive and non-reproductive (migration, parent-young interactions, schooling and related social behaviours) behaviours of fish (reviewed in [1,2]). In general, pheromones are released into the water via the urine, gills, skin or faeces, and they trigger adaptive physiological and behavioural responses in conspecifics. Reproductive hormones (steroids and prostaglandins or their metabolites) released into the water can be employed as hormonal pheromones, reflecting the reproductive status of the donor and affecting the reproductive physiology and behaviour of receivers [2]. Most studies, however, have focused on the role of pheromones in regulating the interaction between the sexes and few studies have dealt with the roles for putative pheromones in the regulation of competitive interactions within sexes. In goldfish (*Carassius auratus*), which have the best-understood sex pheromone system among teleosts [2,3], hormonal pheromones from females function primarily as timing cues to synchronize male and female spawning. In addition, it has been shown that male goldfish respond strongly to the presence of male conspecifics. They increase sperm stores either in response to nearby males with higher levels of the sex steroid hormone 17,20β-dihydroxy-4-pregnen-3-one, or in response to isolation from a group of conspecific males in a basal endocrine condition [4,5]. The latter is probably because of the action of inhibitory male steroidal cues dominated by androstenedione [2], which is released in large amounts through the gills of sexually active males and has been suggested to be a male pheromone regulating competitive interactions among males [6]. In zebrafish (*Danio rerio*), male pheromones stimulate female reproduction and increase the quality and viability of eggs. However, putative pheromones from conspecific females inhibit female spawning[7].

Although the interrelationships between social behaviour (not directly involved in reproduction) and physiology have been extensively studied in several teleost species [8-11], the role of pheromones in the regulation of social behaviour has received little attention. In species where males aggregate in breeding arenas (lek breeders) and dominance hierarchies are established, there is a possibility for chemical communication and a role for pheromones in the modulation of aggressive interactions among males. The Mozambique tilapia (*Oreochromis mossambicus*) is a good model candidate to test this hypothesis because males practice continuous lekking [12], without leaving their territories to forage, during the entire breeding period.

*O. mossambicus *is a maternal mouth-brooder cichlid endemic to the lakes and rivers of the east coast of Africa [13], in which the males form dense aggregations over sandy substrates during the breeding season [13,14]. Territorial males adopt a characteristic black colouration and defend small territories centred on pits (nests) that they dig in the sand. Visiting females spawn in these territories, but brood the fertilized eggs and subsequent fry away from the leks [13,15-17]. The social behaviour of this species has received considerable attention, as has the causal processes underlying colouration patterns (see [18-23] and references therein). In captivity, males form dominance hierarchies which are stable for several days [18,23-25]. The largest alpha males receive more visits by females [25] and the majority of spawnings [18], and the dominance structure is positively correlated with urinary sex hormone levels [19,23]. During hierarchy formation, the initial assessment includes asymmetrical behavioural displays that can escalate to higher levels of symmetrical aggression, including mouth-to-mouth fighting. Once the hierarchy is established, the overall level of aggression decreases and asymmetrical displays are the most common agonistic interactions observed. A further decrease in aggression occurs after the addition of females to the group [23]. During paired interactions 'resident' males dramatically increase their urination frequency in the presence of 'intruder' males [26,27]. Since urine from males is a vehicle of odorants, at least for conspecific females [28], it is possible that dominant/territorial males actively advertise their aggressive motivation and social status to other males using urinary odorants, which may act as male-male pheromones, in addition to visual displays. The goal of the current study was to determine (i) whether the male urination pattern is linked to aggressive behaviour and (ii) whether the olfactory potency of male urine on conspecific males is related with the social status of the male donor.

## Results

### Urination and male behaviour

**Figure 1:**
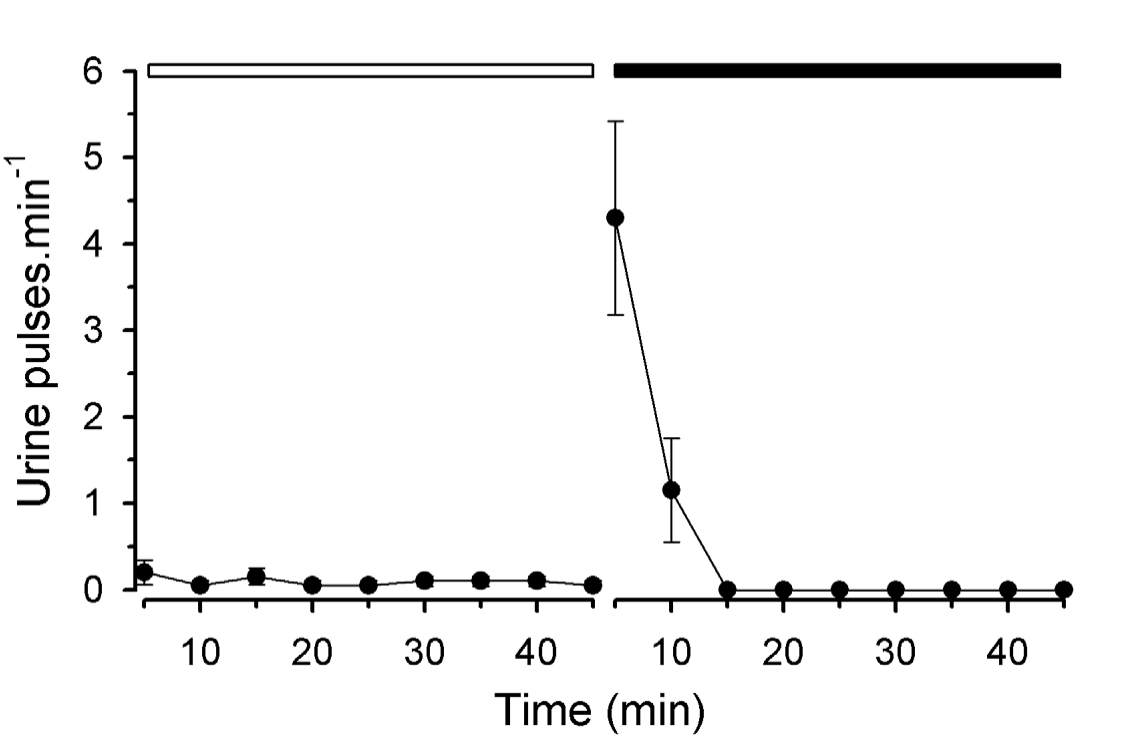
**Urination frequency of male tilapia**. Frequency of urination (pulses per minute every 5 min; mean ± SEM; *n *= 4) by dye-injected resident males in social isolation (open horizontal bar) followed by the presence of a saline-injected intruder male of similar size (dark horizontal bar).

During social isolation male tilapia urinated at very low frequency (on average one pulse every 10 min) and expelled urine in short-duration pulses (less than 2 s). Resident males markedly increased their urination frequency during the first 5 min following the introduction of an intruder. During the subsequent 5 min, the urination frequency decreased and after 10 min resident males stopped releasing urine (Figure 1). All four resident males reacted aggressively soon after the intruder male was introduced into the tank; an increase of urination frequency occurred when resident males initiated aggressive displays. In three cases, resident males eventually stopped releasing urine pulses after the aggressive interaction escalated to mouth-to-mouth fighting. In one case, the intruder male was never aggressive and the resident male eventually stopped the release of urine pulses. Urine release was never observed in resident males whilst being not aggressive. The mean urine pulse duration was 1.4 ± 0.1 s (mean ± SEM, *n *= 126 pulses) and, apparently, the mean pulse duration per male did not change between social isolation (1.4 ± 0.2 s, *n *= 4) and during interaction with the intruder male (1.5 ± 0.3 s), indicating that the total urine volume released increased with urination frequency. No obvious changes in the release of faeces or intestinal fluids were noted. The trend indicating a link between increased urination and the aggressive behaviour of the sending male (without obvious changes in duration of urine pulses or release of faeces or intestinal fluids) was also observed when four male pairs were repeatedly used (after at least a one-week interval). The same male was used four times and in one-half of the replicates was either a dye-injected resident or a dye-injected intruder. However, given that our experimental design did not allow control over possible memory effects of the previous interaction on subsequent male behaviour and urination frequency, we have not included these data.

**Figure 2:**
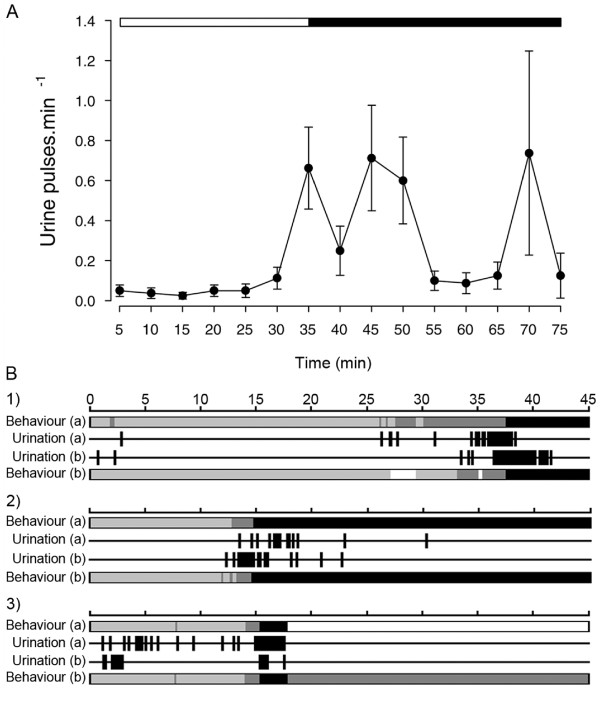
**Urination frequency and behaviour of two neighbouring tilapia males**. (A) Frequency of urination (pulses per minute every 5 min; mean ± SEM; *n *= 16) by males in social isolation (open horizontal bar) followed by contact with a neighbour male (dark horizontal bar). (B) Examples (1–3) of behaviour (submissive: white; not aggressive: light grey; aggressive displays: dark grey; highly aggressive: black) and release of urine pulses (urination), of around 1 s, during 45 min of interaction between two territorial male tilapia (a) and (b). In (1), male (a) increased its urination frequency 25 min after coming into contact with male (b) and initiated aggressive displays which escalated to symmetrical high aggression (circle fight); in turn, male (b) changed from submissive, not urinating, to aggressive displays and its urination frequency increased as the agonistic interaction escalated to high symmetrical aggression. In (2), both males increased their urination frequency within 10–15 min when both initiated aggressive displays which further escalated to high symmetrical aggression; although the two males maintained this level of aggression throughout the observation period, their urination frequency dropped significantly. In (3), we see the only case of high urination frequency observed during non-aggressive behaviours, before aggressive behaviour escalation, during which the frequency of urination was also high; male (a) became submissive within a few minutes after the symmetrical high aggression and stopped urinating, whereas male (b) continued with aggressive displays and stopped urine release as well.

**Figure 3:**
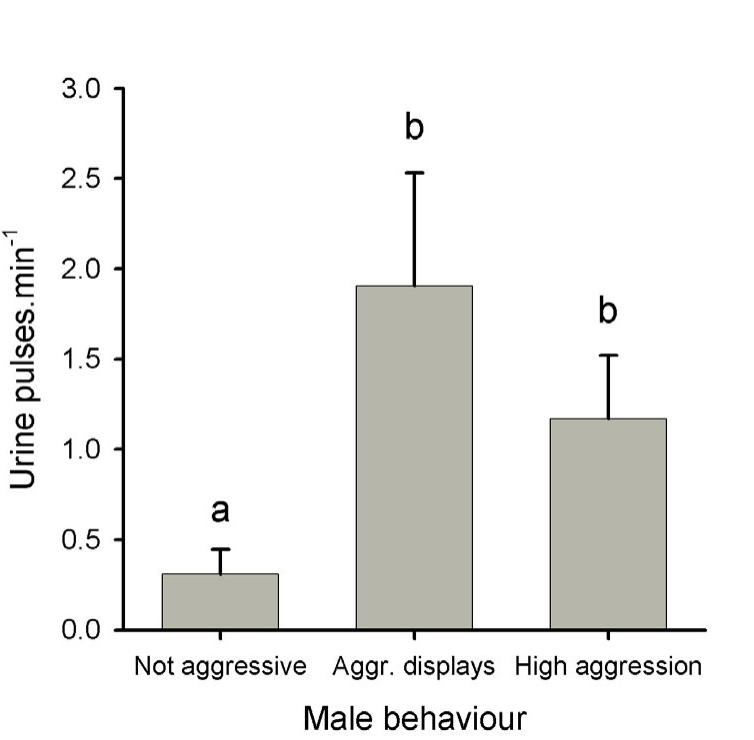
**Urination and behaviour of neighbouring tilapia males**. Urination frequency (mean ± SEM, *n *= 16) during non-aggressive behaviour, aggressive displays and high aggression. Different letters over the bars indicate significant differences (repeated-measures analysis of variance (ANOVA), *F*_1,15 _= 125.6, *p *< 0.001 followed by least significant difference (LSD) pairwise comparisons, *p *< 0.05).

In the experiment with neighbour males, the mean urination frequency increased tenfold when the two males came into contact (social isolation: 0.04 ± 0.02 pulses.min^-1^; with contact: 0.4 ± 0.1 pulses.min^-1^, *n *= 16, Student's *t *test for paired samples, *t*_15 _= 7.43, *p *< 0.001; Figure 2A). The urine volume collected from each male at the end of each observation varied between 0 and 1.6 ml; males from which no urine was collected had high urination frequency during the observation period. In every observation, both males increased their urination frequency, but the level and timing of the increase varied within and between replicates and were related to the level of aggression (Figure 2B provides three examples). In all cases, there were periods of symmetrical aggression during which both males increased urination frequency. Urine release was not observed during submissive behaviours or whenever one male became submissive to its opponent (50% of the replicates). The dominant male also stopped urinating during its aggressive displays when the opponent male became submissive, despite still having urine in the bladder (this was checked at the end of the experiment). Overall, urination frequency was significantly higher during aggressive displays and symmetrical high aggression than during non-aggressive behaviours (Figure 3).

### Social hierarchies, urine volume and olfactory potency of male urine

**Figure 4:**
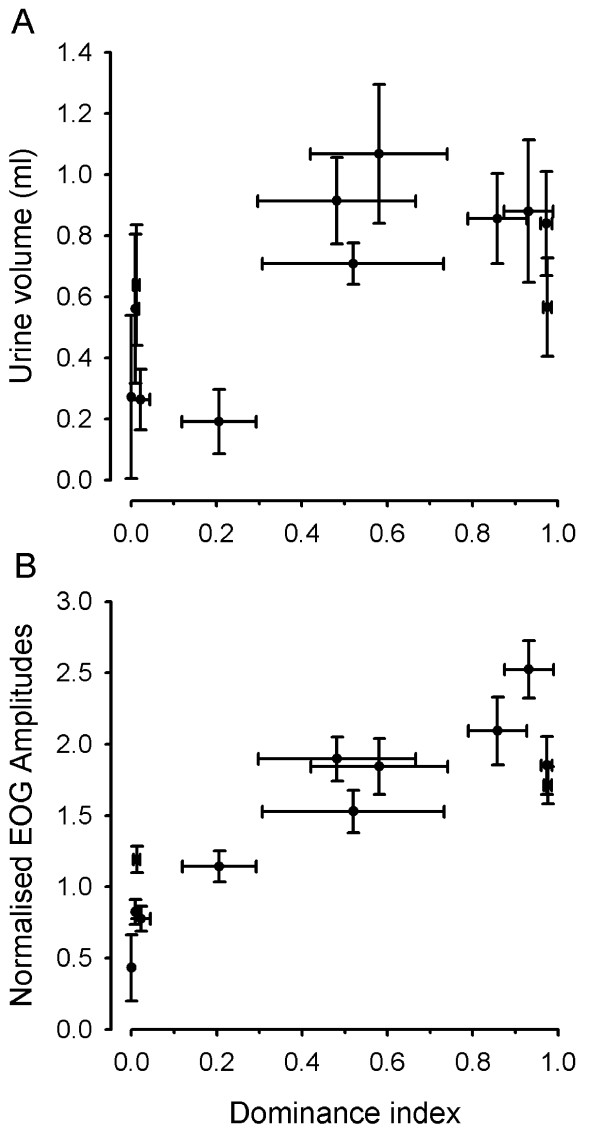
**Urine volume, urine olfactory potency and dominance index of donor male tilapia in a community tank**. (A) Scatter plot of urine volume (mean ± SEM, *n *= 5 days) collected from males (*n *= 12) and their Di (mean ± SEM, *n *= 5 days) during 5 days in a community tank with females. The mean urine volume collected from the five subordinate males (Di between 0.0 and 0.21) was significantly smaller (0.39 ± 0.09 ml) than that collected from males of higher social rank (0.83 ± 0.06 ml; Student's *t *test, *t*_10 _= 4.330, *p *< 0.005). (B) Scatter plot of EOG amplitudes (mean ± SEM, *n *= 30) elicited by male urine (1:10^4 ^v/v in water) and the dominance index (mean ± SEM, *n *= 5 days) of donor males (*n *= 12). The urine of subordinate males evoked significantly smaller normalized EOG responses (0.87 ± 0.14) than that of higher rank males (1.92 ± 0.12; *t*_10 _= 5.715, *p *< 0.001).

In the community tank, five males had a subordinate status (daily dominance index (Di): 0–0.21) and seldom or never exhibited dominant behaviours. The other seven males were of higher social rank (Di ≥ 0.5); four of the high social rank males were the most dominant males (Di: 0.73–0.91), occupied a fixed position in the tank and seldom or never showed submissive behaviours. Size and growth rate was not different between subordinate males (standard length (SL) = 148.2 ± 1.7 mm; body weight (BW) = 96.8 ± 3.6 g; SL growth = 0.18 ± 0.04 mm.day^-1^; BW growth = 0.29 ± 0.13 g.day^-1^) and males of higher social rank (SL = 152.9 ± 2.0 mm, *t*_10 _= 1.697, *p *= 1.121; BW = 109.1 ± 5.3 g, *t*_10 _= 1.771, *p *= 1.107; SL growth = 0.18 ± 0.05 mm.day^-1^, *t*_10 _= 0.037, *p *= 0.971; BW growth = 0.28 ± 0.14 g.day^-1^, *t*_10 _= 0.055, *p *= 0.958). However, the mean urine volume collected from subordinate males was lower than that from males of higher social rank (Figure 4A). Also, the mean electro-olfactogram (EOG) amplitude evoked by urine from subordinate males was significantly smaller than that elicited by urine samples from males of higher social rank (Figure 4B).

## Discussion

The current study provides strong empirical evidence for a close link between male aggression and increased urination in male tilapia. This behaviour is different from that reported in earlier studies [26,29], in which males increased their urination frequency when a pre-ovulatory female was introduced into their tank. A major difference in the response to pre-ovulatory females, however, is that the increase of urination frequency was not linked to specific behavioural displays (e.g. courtship) whereas in the current study the increase of urination frequency occurred together with aggressive behaviours. Pre-ovulatory female tilapia release specific odorants into the water and males base their increase in urination frequency on this olfactory information which does not affect the intensity of courtship behaviours [29]. It is not known whether odorants from conspecific males may modulate the behaviour and/or urination frequency as suggested for male-female interactions.

In all cases when both males released urine during symmetrical aggression without the emergence of a loser or winner, high urination frequency occurred immediately before the aggression or soon after the start of aggressive displays. Also, in all cases when a male soon became submissive, the aggressive male did not release urine. When a male became submissive after engaging in symmetrical aggression, the dominant male stopped urinating. As the urine is a vehicle of potent odorants, especially urine from males of higher social rank, and urine was never released during submissive behaviours, these results raise the possibility that aggressive territorial males actively release chemical information to rival males. Urinary odorants, alone or in a mixture with possible and additional odorants from other sources (e.g. skin mucous, faeces and/or gills), may reinforce information about the aggressive motivation of the sender conveyed through other sensory channels such as vision (sound is not emitted during aggressive behaviour [30]). However, the precise effect that urinary odorants may have on the receiver is not yet clear. Possibly, depending on its motivational state and the opponent's behaviour, a male may give up the dispute; the decision-making process could be influenced by chemical information in its opponent's urine. Only manipulative experiments will show the effect of urine on receiver males and whether and how urine odorants are necessary to exert a dominant status.

The identities of the urinary odorants are as yet unknown. In the tilapia, the concentration of urinary steroids depends on social context. Testosterone (T), 11-ketotestosterone (11KT), 17,20β-dihydroxy-4-pregnen-3-one (17,20β-P) and 17,20α-dihydroxy-4-pregnen-3-one (17,20α-P) are positively correlated with dominance [19]. However, none of these steroids evoke significant EOG responses even at concentrations as high as 10^-6 ^M (see [28]). Some empirical evidence suggests that, at least in some species, gonadal androgens may function as male-derived pheromones that denote maturational state or gender. In goldfish, sexually active males release, via the gills, relatively large amounts of androstenedione [6] which stimulates pushing behaviour (a component of male-male competition) [31] and has been suggested to act as a male pheromone (as part of a blend of steroids dominated by androgens) in the regulation of competitive interactions among males [2,6,31]. Mature male silver barbs, *Punctius schwanenfeldi*, detect 11KT at threshold concentrations of about 10^-11 ^M (see [32]). In the lek-breeding African cichlid, *Haplochromis burtoni*, both sexes appear to detect only conjugated (sulphate or glucuronide) sex steroids (at thresholds of 10^-11 ^M) of which six were androgens including testosterone-sulphate (T-17S) [33]. Although the olfactory system of the Mozambique tilapia is not sensitive to T, T-17S or 11-KT [28], other androgen metabolites in male urine, which may also correlate with dominance, could act as odorants. However, a recent study using liquid chromatography linked to mass spectrometry and recording of EOG showed that female tilapia detect a single compound in male urine, suggested to be a sulphated amino-sterol (Barata et al., unpublished). This odorant is present at higher concentrations in the urine of dominant males than in subordinate males. Whether this is the same and only odorant in male urine that is detected by conspecific males is under investigation.

The current study shows that dominant males actively release chemical information by increased urination during aggression and that their urine has higher olfactory potency than that of subordinate males. Although these results support a role for urinary odorants in advertising aggressive motivation and dominance, other odorants are released to the water via intestinal fluids and, possibly, the gills and skin [28]. These may also reflect male social status and have a role in mediating aggressive interactions among males. However, no obvious changes were seen in the rate of release of intestinal fluids. Nevertheless, whether urinary odorants are the most important odorants or whether other odorants released via other routes are also involved in male-male interactions needs further investigation.

In contrast, females release urine in shorter pulses, but at higher frequency, than males. Furthermore, this is apparently unaffected by the presence of a sexually active male [26]. Since a territorial male of 100 g can accumulate up to 2 ml of urine in the urinary bladder [26], storage of urinary odorants for release in the appropriate social context seems to be a male-specific characteristic for this species. On the other hand, increase of urine release in association with visual displays could represent a simple modification of an extant behaviour to convey and amplify chemical information that adds to visual displays, and to sound, in defined social contexts, i.e. territorial disputes or courtship. As the duration of pulses did not change with increased urination frequency, the total volume of urine released should also increase. In addition, a pulsed signal creates intermittent stimulation and is less likely to cause sensory adaptation in the receiver, especially in the close proximity that these social interactions occur.

Urine volume collected from dominant males was consistently higher than that collected from subordinate males. Although it could be argued that the method used to collect urine does not allow for accurate estimates of urine volume in the bladder, the fact that it was employed consistently by the same person and that care was taken to randomize the order by which males were sampled suggest that our measurements are valid for the relative comparison between males of different social rank. Furthermore, difference in stored urine volume between dominant and subordinate males has been observed in two other social groups (with the same number of fish in similar tanks) sampled for urine in the same way (Barata et al., unpublished). Taken together, these results suggest that social dominance may require controlled release of urine during the appropriate social interactions and that this ability is less apparent in subordinate males and females.

Freshwater fish are hyperosmotic to their environment and need to expel excess water to maintain osmotic balance and, therefore, tend to produce large volumes of dilute urine. The outlet through the urogenital papilla is guarded by one or more sphincters, and in freshwater rainbow trout the urine can be stored in the bladder for about 25 min before release (periodic pulses at 20–30 min intervals) [34]; this storage period seems to allow the bladder epithelium to supplement the kidney in reabsorption of NaCl [34,35]. Social status may affect the capacity/need to control urine storage and release from the urinary bladder; subordinate males may produce less urine (lower glomerular filtration rate), dominant and subordinate males may produce similar volumes of urine but subordinates retain lower volume in the bladder, or both urine flow rate and storage capacity are different between subordinate and higher rank males. To the best of our knowledge, these questions have not yet been addressed in the tilapia. In rainbow trout, urine flow rates and plasma cortisol concentrations are higher in subordinate fish but there is no difference in glomerular filtration rate between dominant and subordinates [36]. By extension, if social subordination induces increased urine flow in tilapia, then the larger urine volume collected from dominants could be explained by an active and precise control of release of urine stored by dominant males.

The physiological mechanisms that control the urination behaviour of fish have not been studied. In mice, however, changes in androgen levels affect the urinary tract and urination behaviour. Testosterone induces increased bladder muscular mass and affects the urination pattern of both males and females [37]; male mice deposit urine around territorial boundaries, through scent-marking behaviour, to advertise territorial dominance and their competitive ability to potential mates and competitors, also allowing individual recognition [38,39]. In male tilapia, androgen levels (11KT) are good predictors of dominance [19], but their effect in urinary tract physiology or urination behaviour is not known. Mature masu salmon show morphological changes in the urinary tract suggesting a functional transition in the kidney related to the release of sex pheromones in both sexes [40,41]. Further investigation is necessary to determine which physiological mechanisms (e.g. urinary bladder morphology and neuroendocrine control of urine storage and release) explain the different urination behaviour between subordinate and dominant males, and how a shift in social status may affect such mechanisms in the tilapia.

Urinary odorants may be involved in the emergence of social hierarchy at the initial stage of lek formation and, thereafter, in the maintenance of social stability as previously described [18,23-25]. Once a male has gained a high social status, maintaining dominance may result, at least in part, from its ability to respond to challenges from other males with urine release that reflect its physiological state. On the other hand, olfactory input during previous disputes may affect the pattern of subsequent social interactions. In the tilapia, winning or losing an encounter with another male modifies subsequent sound production and courtship [42]. However, it is not yet known whether urinary odorants play a role in this effect or if previous fighting experience affects the urination behaviour.

In general, fighting experience influences the outcome of a later contest (known as the winner and loser effects) and may mediate the formation of social hierarchies where individual recognition might reduce aggression among group members (reviewed in [43]). In the closely related Nile tilapia, which has a similar reproductive strategy, fish holding-water decreased aggression within pairs of juvenile fish and this was more accentuated when the fish pairs (of undetermined sex) were from the same original group [44]. Undoubtedly, individual recognition is an important stabilizing factor for social hierarchies in permanent or semi-permanent social groups [45,46] and fish are capable of individual recognition through olfaction [47] and through associative learning with olfactory cues [48]. In the cichlid, *Astatotilapia burtoni*, which have reproductive and social systems similar to those of tilapia, males seem to build spatial and featural representations related to rival abilities which they can use to infer social rank [49]. It is possible that the build up of memory from such visual information is aided by olfactory input allowing further individual recognition and the assessment of a rival's social rank. Based on our study, we suggest that urinary odorants may play a role in the above-mentioned aspects of male tilapia social behaviour, although further investigation is required.

The release of urine signals during aggressive interactions may not be unique to male tilapia and may be found in fish that aggregate temporarily and/or fight for territory, having high levels of intra-sexual competition. To the best of our knowledge, however, this has not been investigated, but it is certainly not unique to fishes. In some crustaceans, increased release of urine occurs during aggressive interactions between males [50-52], suggesting that linking urine signals to aggressive behaviour may have evolved independently in different taxa of aquatic animals.

## Conclusion

Dominant males store more urine in the bladder than subordinates. Urine is actively released when the male is involved in aggressive disputes. The olfactory potency of urine from dominant males is greater than that from subordinates. We suggest that urine is a vehicle for male-pheromones which may aid in exerting social dominance.

## Methods

### Experimental animals

Mozambique tilapia were obtained from a brood-stock maintained at the University of Algarve (Faro, Portugal). For several weeks before use in experiments, groups of fish (one male and three to four females) were housed in re-circulating aquaria (93 × 60 × 55 cm) with sand substrate, containing 200 l de-chlorinated tapwater at 27°C, photoperiod 12 L : 12 D. Fish were fed twice a day with a commercial cichlid food (Nutrafin basix^®^; Rolf C. Hagen, Inc., Montreal, Canada). Spawning occurred in all fish groups, but the fertilized eggs were removed and incubated elsewhere.

### Visualization of urination

Urination was visualized as described previously [26], using a method based on that of Appelt and Sorensen [53]. Males were lightly anaesthetized by immersion in iced water for 2–3 min and injected in the dorsal musculature with 100 μl of 100 mg.ml^-1 ^patent blue violet (Sigma) in 0.9% NaCl. The fish were placed back in their tank, allowed to recover and experiments started after the first visible pulse of blue urine. Although fluids released through faeces are also coloured, urine seen was as a blue plume jetting from the urogenital opening in the genital papilla and was clearly discernible from release of faeces or fluids from the anus.

### Urination and behaviour in male-male interactions

A first set of experiments was carried out to assess urination frequency and duration in social isolation and during aggressive interactions. The resident-intruder paradigm was employed with four pairs of size-matched territorial males (SL = 125.6 ± 3.6 mm, mean ± SEM, *n *= 8; BW = 62.3 ± 4.4 g; maximum SL and BW difference between two males was 1 mm and 3.9 g, respectively). The two males were taken from their family tanks and isolated in glass tanks (80 × 35 × 45 cm, 50 l, 27°C, aerated) with sand substrate for 24 h prior to the experiment. The resident male was injected with the dye and the 'intruder' male was injected with saline (0.9% NaCl). After 45 min in social isolation, the resident male received the intruder into its tank and the behaviour of both fish was recorded on video for 45 min (Panasonic SX60 super VHS). Urine release and duration of each urine pulse was recorded by the observer using a handheld computer (Psion Organizer LZ64) programmed for timing start and end of each urine pulse through the stroke of two keys. Immediately after each replicate, the males were lightly anaesthetized by immersion in iced water for 2–3 min and urine was sampled by gently squeezing the abdomen immediately above and anterior to the genital papilla. Squeezing of this abdominal area caused erection of the genital papilla and a jet of urine was collected. Urine volume was measured by weighing. Each male pair was used only once and, in each replicate, only the 'resident' male was injected with the dye.

In the second set of experiments, the neighbouring males paradigm was used. Eight pairs of size-matched males (SL = 136.6 ± 3.2 mm; BW = 76.8 ± 5.0 g; maximum SL and BW difference between two males was 1 mm and 4.1 g, respectively) were chosen. Two males were injected with the dye and placed in either side of a glass tank (similar to the method described above), separated from each other by a sliding opaque partition in the middle which prevented visual or chemical contact between the two sides. After recovery, the males were filmed for 30 min. The partition was then lifted and filming continued for another 45 min. The frequency of urine release was recorded by the observer using the handheld computer programmed for timing urine release from each male. Immediately after each replicate, the males were sampled for urine and returned to their original family tanks.

The Observer PC software V4.0 or V5.0 (Noldus Information Technology, Wageningen, The Netherlands) was used to score the frequency and duration of male behaviours recorded in the videos. The behaviours were grouped into the following behaviour classes: non-aggressive behaviours (e.g. swimming), submissive behaviours (escape from an aggressive opponent or submission postures), aggressive displays and symmetrical high aggression (biting or chasing each other in a circle interrupted by mouth-fighting). These behaviours are described in detail by Baerends and Baerends van Roon [16] and Oliveira [22].

### Social hierarchies in a community tank

To establish social hierarchies and obtain urine samples from dominant and subordinate males for subsequent assessment of olfactory potency on males (see below), 12 males and 14 females (all sexually mature) were housed for 23 days in a community white plastic tank (128 × 110 × 50 cm) containing de-chlorinated tap-water (500 l) at 25–27°C, photoperiod 12 L : 12 D, and feeding was once a day with commercial cichlid food. The males were tagged with coloured plastic labels (T-Bar extra small anchor, FF-94, FLOY TAG Inc., Seattle, WA) attached to the muscle near the dorsal fin and their behaviour was systematically observed during three observation sessions per day (morning, midday and afternoon) over 5 days starting 18 days after group formation. In every observation session, the group was observed continuously for 60 min and the frequency of submissive behaviours (escape from an aggressive opponent or submission postures without dark colouration), dominant behaviours (aggressive displays or nest-digging) and courtship displays were recorded for each male. Although the community tank had no sand, some males exhibited normal nest-digging behaviour by nipping the bottom. After the afternoon observations, each male was caught with a net, slightly anaesthetized (50 mg.l^-1 ^3-aminobenzoic acid ethyl ester, MS222) and sampled for urine as described above. The fish were allowed to recover and placed back in the tank. The urine from each male was stored at -20°C until use for the assessment of olfactory potency (see below). To quantify the males' social rank, Di was calculated as the ratio between the summed frequency of all dominant behaviours (including courtship) and the summed frequency of dominant and submissive behaviours. The SL and BW of the males were measured at the start of the social group formation (SL = 146.8 ± 1.6 mm, BW = 97.3 ± 3.8 g) and at the end of the third week after group formation (SL = 150.9 ± 1.5 mm, BW = 104.0 ± 3.7 g). During the 3 weeks, males had a small but significant growth in length (0.18 ± 0.03 mm.day^-1^, *t*_11 _= 5.74, *p *< 0.001) and weight (0.29 ± 0.09 g.day^-1^, *t*_11 _= 3.06, *p *< 0.05).

### Recording of the EOG and stimuli preparation

To assess the olfactory potency of urine from males of different social status, EOGs were recorded in males as described previously [28]. Briefly, male tilapia were anaesthetized in water containing 100 mg.l^-1 ^MS222 and immobilized with gallamine triethiodide (3 mg.kg^-1 ^in 0.9% saline). The fish were placed in a padded Perspex^® ^V-clamp with their gills irrigated with aerated water containing MS222 (50 mg.l^-1^). The olfactory rosette was exposed by removing the ring of cartilage surrounding the nostril and continually irrigated with de-chlorinated, charcoal-filtered tapwater (6 ml.min^-1^). Stimulus-containing water was introduced into this flow via a three-way solenoid valve. At least 1 min was allowed to elapse between successive stimuli. The DC voltage was recorded by two glass micropipettes filled with 0.9% NaCl in 4% agar, one being placed close to the olfactory epithelium and the other placed lightly on the skin of the head. The signal was amplified (×10^3^) and recorded on a PC running Axoscope software (version 1.1, Axon Instruments, Inc., Foster City, CA). The peak amplitude of the EOG response to male urine was blank-subtracted and normalized to the response to 10^-5 ^M L-serine (standard). At the end of each recording session the fish were killed with a sharp blow to the head and measured for size and weight of body and gonads. All stimuli were prepared on the day of EOG recording using the same de-chlorinated, charcoal-filtered tapwater used to irrigate the fish's nostril. To test for a relationship between the olfactory potency of male urine (at a dilution of 1:10^4 ^in water) and the social rank of the male donor, urine taken daily from each male in the community tank was tested (60 urine stimuli) on the same male and the procedure was replicated with six males testing the samples from each donor male in random order (SL = 149.7 ± 5.6 mm; GSI = testes weight/body weight × 100 = 0.75 ± 0.07%).

### Statistical analysis

The Student's *t *test for paired samples was used to compare the urination frequency of neighbouring males between social isolation and during the aggressive interaction. Repeated measures analysis of variance followed by the least significant difference (LSD) pairwise comparisons test were used to compare urination frequency (values transformed by log (*x *+ 1.5)) during non-aggressive behaviours, aggressive displays and high aggression by neighbouring males. The mean urine volume collected per male, mean EOG amplitudes elicited by male urine, male size and growth were compared between five subordinate males (Di between 0 and 0.21) and seven dominant males (Di between 0.48 and 0.98) using the Student's *t *test for independent samples. All data are shown as means ± SEM and statistical significance was established at *p *< 0.05.

## Authors' contributions

ENB devised the study, participated in the planning of all experiments, analysed the behavioural data collected with the Observer Video-Pro, performed all statistical analyses, participated in the discussion of results and wrote the manuscript. PCH participated in the conception and design of the study, planning of all experiments, supervised the recording of EOGs, contributed to discussion of results and wrote the manuscript. OGA planned and carried out the behavioural experiments with the male pairs, collected behavioural data with the Observer Video-Pro and participated in analysis and discussion of results. AM planned and carried out the behavioural observations of the males in the community tank and the recording of EOGs and contributed to statistical analysis and discussion of the results. AVMC provided resources, contributed to discussion of results and wrote the manuscript. All authors read and approved the final manuscript.
